# Learning-by-Concordance (LbC): introducing undergraduate students to the complexity and uncertainty of clinical practice

**Published:** 2016-10-18

**Authors:** Nicolas Fernandez, Amélie Foucault, Serge Dubé, Diane Robert, Chantal Lafond, Anne-Marie Vincent, Jeannine Kassis, Driss Kazitani, Bernard Charlin

**Affiliations:** 1 Department of Family Medicine and Emergency Medicine, Faculty of Medicine, Université de Montreal; 2 University of Toronto, ON; 3 Faculty of Medicine, University of Montreal; 4 Emerge Inc., Montréal, QC

## Abstract

**Background:**

A current challenge in medical education is the steep exposure to the complexity and uncertainty of clinical practice in early clerkship. The gap between pre-clinical courses and the reality of clinical decision-making can be overwhelming for undergraduate students. The Learning-by-Concordance (LbC) approach aims to bridge this gap by embedding complexity and uncertainty by relying on real-life situations and exposure to expert reasoning processes to support learning. LbC provides three forms of support: 1) expert responses that students compare with their own, 2) expert explanations and 3) recognized scholars’ key-messages.

**Method:**

Three different LbC inspired learning tools were used by 900 undergraduate medical students in three courses: Concordance-of-Reasoning in a 1^st^-year hematology course; Concordance-of-Perception in a 2nd-year pulmonary physio-pathology course, and; Concordance-of-Professional-Judgment with 3rd-year clerkship students. Thematic analysis was conducted on freely volunteered qualitative comments provided by 404 students.

**Results:**

Absence of a right answer was challenging for 1^st^ year concordance-of-reasoning group; the 2^nd^ year visual concordance group found radiology images initially difficult and unnerving and the 3^rd^ year concordance-of-judgment group recognized the importance of divergent expert opinion.

**Conclusions:**

Expert panel answers and explanations constitute an example of “cognitive apprenticeship” that could contribute to the development of appropriate professional reasoning processes.

## Introduction

The educational literature is rich in studies that expound the importance of context on learning, especially the interventions of teachers and their interactions with students. Cognitive apprenticeship studies have focused on the role of teachers – as role models – in providing meaning and structure to learning,^1^,^2^ namely by modeling their professional practice.^3^,^4^ A central premise of cognitive apprenticeship^5^ is that instructors should purposely reveal their mental processes in order to provide effective support for development of problem solving skills in their students.^6^ Such an approach to teaching has been shown to favor complex skill development and motivation in higher education.^7^–^9^

### Learning by Concordance

Learning by Concordance (LbC) leverages the benefits of information technology to provide students with cognitive tasks embedded in real-life clinical situations and expert reasoning processes to solve them.^10^,^11^ We tested three formats of online LbC in three North American undergraduate medical curriculum courses, for differing cognitive tasks and at different undergraduate training levels: 1) Concordance-of-Reasoning in a first-year hematology course, 2) Concordance-of-Perception in a 2nd-year pulmonary physiology course and 3) Concordance-of-Professional-Judgment with 3rd-year clerkship students. The aims of this article are to (1) describe the method with its common and variable features and (2) describe the advantages and limitations of this innovative learning method as conveyed by undergraduate students who used it.

### Description of LbC tools

#### A. Common features

In all LbC tools, a real-life clinical situation is described briefly, in a short statement, a radiographic scan or graph. A question a professional would entertain in that situation is presented (e.g. interpreting blood analyses, reading radiographic scans). Participants’ answers are registered on Likert scales (for reasoning and judgment tasks) or by circling or pointing out the abnormalities on a screen (e.g. on a radiographic scan). Once students submit their response, targeted feedback by experts is generated. The feedback includes justifications given by the experts about their responses and key-messages provided by a recognized scholar in the field, including hyper-links to complementary resources (e.g. scientific paper, website, etc.). Expert responses may reflect various relevant solutions to the same situation.

#### B. The variable features

The different types of learning by concordance are differentiated by a) the nature of the task the student must complete, b) the means of capturing student responses and c) the number and kind of experts.

##### a) Nature of the task

###### Clinical reasoning task

In concordance-of-clinical reasoning, the task is embedded in script theory.^12^,^13^ It consists of interpreting the significance of key data experienced clinicians seek out and use to intervene (Table 1). Hence, the student can compare their own reasoning with that of the members of the expert panel.

###### Ethics and professionalism judgment task

In the concordance-of-judgment, the student is placed in a situation where common professionalism issues of clinical practice are involved.^14^,^15^ Different behaviors relevant to the given situation are presented. The task consists of deciding the degree of appropriateness for each behavior. Table 2 presents the screens seen by the student. The example shows the diversity of explanations given by experts and their justifications.

###### Perception and interpretation on medical images

**Figure 1:**
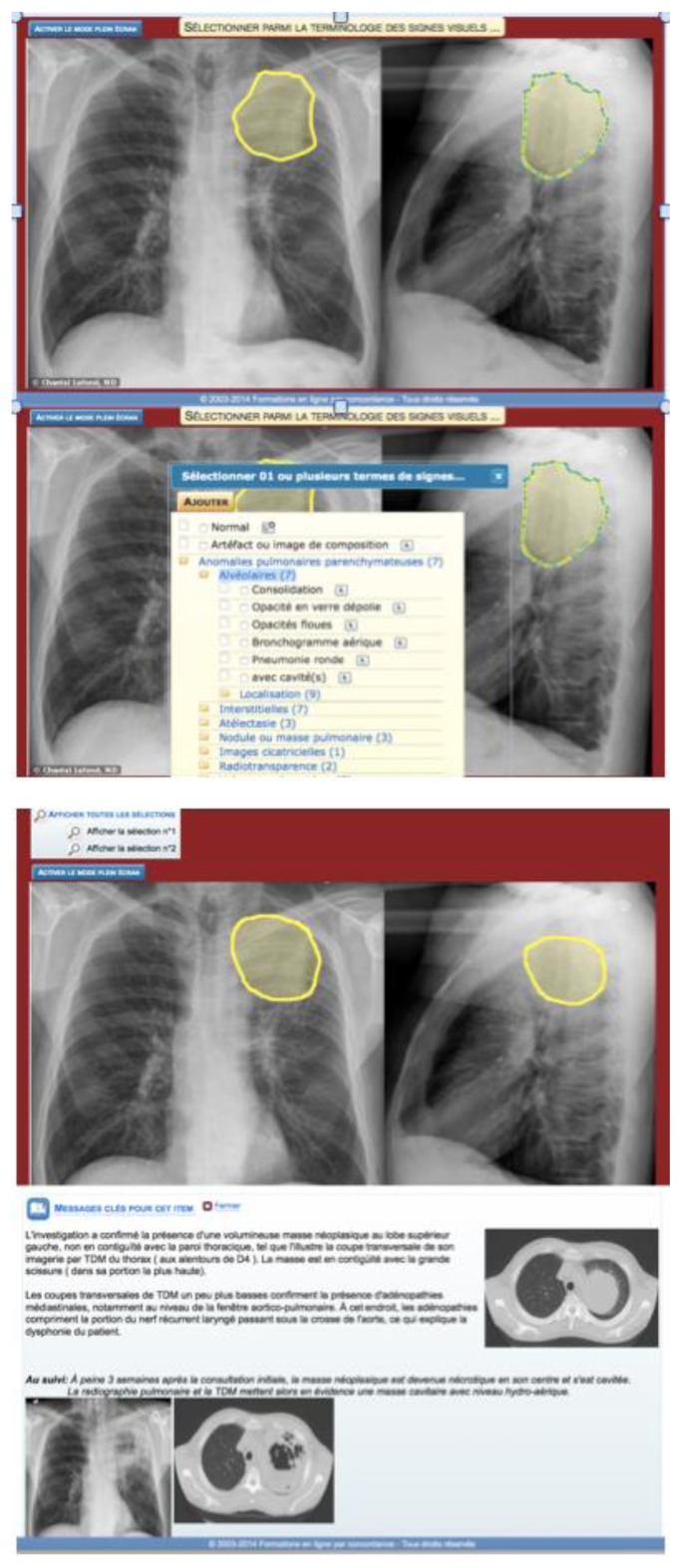
Succession of screens (from top to bottom: Participant delineates the perceived lesion area, then categorizes it (semiology), then participant discovers the delineation provided by the teacher followed by useful complementary information)

In fields of medicine where images are diagnostic tools (radiology, pathology, dermatology or ophthalmology), it is sometimes difficult to know if the students truly see what the instructor is showing them and whether they are interpreting the images correctly. Learning by concordance-of-perception addresses this issue by asking the students to detect and interpret abnormalities that appear on the images. A short “vignette” is presented comprising clinical information, with or without cues or signs and patient’s symptoms. Students must draw on radiographs (or any type of image commonly used in clinical settings, EKG, histology section, retinal images, etc.) the abnormalities they detect, interpret and name them. Figure 1 presents an example of the images that are included in this tool.

##### b) The means of capturing student responses

For concordance-of-clinical-reasoning or professional judgment student responses are captured on Likert scales with 3, 4 or 5 levels depending on the nature of the task and students’ training level. Students in a first-year class generally do not have sufficient mastery of concepts to distinguish between “somewhat acceptable” and “totally acceptable.” Clerks, 3rd or 4th year students, are more apt to make such distinctions. Hence, in the example given in Table 1, we used a three-point Likert type scale for first year students, whereas for clerks, we used a four Likert type scale (Table 2). Moreover, in concordance-of-judgment it is desirable to avoid the neutral position (neither unacceptable and neither acceptable), hence the four-point Likert scales forces the student to make a decision. In visual perception concordance, the drawing made around the point of interest captures student response, or by an arrow that student places to point to it or by any other mark on the screen.

##### c) The number and kind of experts

In LbC, the participant gauges his ability through concordance between his response and that of experts in the field. 16 The number of experts depends on the training level of the respondents. For first-year students, explanations given by the course instructor are sufficient. For advanced training levels, (clerkship, residency) it is generally preferred to have several experts, thus reflecting the diversity of perspectives that often occurs in a given clinical situation.

We were interested in finding out whether medical students perceive they can learn by concordance and whether it as a useful tool for their future practice.

## Methods

The three LbC tools presented in this study were tested on 900 students at one of the largest medical schools in Eastern Canada.

### Learning by concordance of reasoning

This tool was tested with a cohort of first year students (*n*=300) as a complementary learning activity in a hematology course taught mainly through Problem-based Learning (PBL). LbC was used to consolidate, through group discussion and debate, newly acquired concepts (see Figure 1). After selecting their response, students accessed course instructors’ responses and explanations on the computer screen. A total of 58 students (19.3% response rate) provided qualitative comments about their experience.

### Learning by concordance of perception

This learning tool was introduced in the pulmonary physio-pathology course for 2^nd^-year medical students and consisted of ten radiographic images. The activity was optional and was completed by the students at times that were convenient for them on their own laptops. The course instructor provided responses and explanations. A total of 241 students took it and 199 provided qualitative comments (83% response rate).

### Learning by concordance of judgment

This on-line learning tool confronted clerks with 20 real-life situations that they are likely to experience during their rotations. These situations included disclosure of medical errors, respect of confidentiality, breaking unpleasant news to a patient as well as regulatory issues and bedside manners.

Panel members were selected by asking all clerks of a cohort to designate three clinical instructors they considered as role models of professionalism. Instructors whose names were the most frequently mentioned were invited to participate. By accepting, they had to respond and explain their responses for all 20 cases. We also requested an expert on professionalism and medical ethics to write key-messages stemming from these kinds of situations. All third-year students (n=300) completed the online exercise on their own computers at home at times most convenient for them. A total of 241 provided qualitative comments (80% response rate).

### Qualitative data collection and analysis

Qualitative data were collected immediately after students completed one of the three concordance learning tools. Students are invited to complete an online evaluation survey after every course in accordance to university policy. For this study, a text window was added to the standard evaluation form to capture qualitative personal comments specifically about the LbC component of the course. The prompting question asked: “Please share any comments about the LbC activity”. The open responses by the students are deemed to adequately reflect students’ perceptions in so far as anonymity was assured and there was no advantage to be gained in participating. The provision of such comments was entirely voluntary; students could easily refrain from leaving any comments if they wished. Table 3 presents the number of students who used the LbC tools and the number of comments that were provided.

We coded and identified common themes embedded in the study material comprised of 404 unprompted comments.^17^,^18^ Analysis was performed in three steps. In step one (initial review), one member of the research team (BC) identified and described themes that emerged from the data set. To limit the effect of our own biases, in step two, all student comments were examined independently by another member of the research team (NF) to label and categorize each extraction until theme saturation was achieved. To complete step two, the research team reviewed the independently created themes and discussed their interpretation according to the research questions. Disagreements were resolved through discussions until a consensus was achieved. In step three, one author (NF) reviewed and coded all quotes for accuracy and consistency.

## Results

### Learning by concordance of reasoning

First year students found the early introduction to uncertainty of clinical practice through LbC challenging but recognized its utility.

*The LbC tool introduces, in fact, uncertainty and ambiguity when we are challenged by a given situation, because other hypotheses can be considered in solving the problem. However, I think the initiation to the LbC from the first year on allows us to develop the judgment required in our future practice.* (No.25M)

*I think it is rather interesting to be introduced to this early rather than continue believing that practicing medicine is like answering multiple-choice questions.* (No.38M)

In particular, the LbC tool allowed students to verify mastery of basic knowledge.

*I appreciated the possibility of completing the LbC tool, it allowed me to see if I had assimilated the knowledge, and if I was wrong, the responses allowed me to gain a better understanding of the material.* (No. 119M)

Students also recognized the value of the tool with regards to critical thinking and reflection.

*It’s by doubting our responses that it is possible for us to have a critical reflection and bolster our learning.* (No.13M)

However, there was a sense, conveyed in some student comments, that the expert explanations were a little confusing. Many students realized they found those questions challenging because they had not yet been exposed to clinical practice.

*Too much, way too much ambiguity and we could argue for every question. Even the responses and the explanations of the instructor weren’t very convincing in some cases.* (No.37M)

*Sometimes “expert” responses were too farfetched in my view and this made it easier to get us mixed up more than anything else. Even if they seemed a little too farfetched, I appreciated having the explanations.* (No.59M)

The absence of the “right answer” was problematic for first-year students. Many comments provided by these students showed unease with the ambiguity in the situations presented. One comment summarizes this:

*The correct answers were not always properly singled out.* (No.2T)

In conclusion, first year students found the LbC tool tested them and bolstered their acquisition of basic hematology knowledge. Also, responding to the vignettes helped them develop skills that would be useful in future practice. Finally, they found the vignettes a little challenging and the expert explanations hard to follow.

### Learning by concordance of perception

Second-year students acknowledged that the visual perception LbC tool is useful to review material and consolidate the knowledge about the course subject matter. It allowed them to identify knowledge gaps. The radiographic images were real and students found it difficult and “unnerving” to attempt to identify pathologies they had not yet “seen” in their curriculum. Many wished they could access the content after having completed the exercise, especially the instructor’s solutions and explanations.

*It would be fantastic to be able to have permanent access to the information even after the training is over, at least till exam time.* (No. 87)

Some indicated that LbC was more effective than large lectures, even though they felt that they would require many more vignettes to become good at radiology and that theoretical teaching beforehand would be useful. The following excerpt summarizes this:

*I think this activity was very interesting. It helped us consolidate our knowledge. By presenting a clinical situation at the beginning, we feel less lost than by just reading the PBL problem statement.* (No.10)

Students appreciated having some kind of guidance from teachers, as witnessed in this excerpt:

*I think that it would be VERY useful to have arrows that point out on the lung the different healthy structures and that there always be a healthy lung next to the pathology so that we can compare. Also, it would be useful that we could have more lectures on radiological images because we are still very far from being good at interpreting them.* (No.50)

Nonetheless, second-year students who undertook the concordance of perception activity agreed that the situations reflected realistic clinical situations they would encounter later in their practice. The following excerpt echoes the sentiment expressed by many comments:

*[The tool is] ideal because I find that it really puts us in a clinical context and it really prepares us for this practice which is not that far off in time.* (No. 55)

In summary, students agreed that the exercise is relevant and useful. They felt that it helped them develop skills for their subsequent practice and found the feedback from their tutors useful.

### Learning by concordance of judgment

Clerks found the professionalism cases presented appropriate for their level of training and, most importantly, forced them to reflect about professionalism issues. Indeed, some mentioned that in the short period of time they had been in clerkship, they had been witness to similar situations to those presented in the LbC tool.

*I find the clinical vignettes VERY useful for clerks who are starting out in the clinical wards. For example, how to react in the face of a conflict with a resident or an attending and how to ask for help when we feel overwhelmed (or when to ask for help).* (No.2)

*The situations are very relevant, realistic and I would have liked to have it before I started clerkship to “avoid professional slip-ups.”* (No.5)

The tool led them through a reflective process that confronted their environment and their behaviors. The idea that this tool provided some guidance for dealing with professional issues, or “avoiding professional slip-ups” as mentioned above, or knowing how to act if ever similar situations arose, was present in many comments. Clerks stated that the situations confronted them with familiar situations and placed them in a position to “exercise our judgment.” (No.65) They were keenly aware that by reflecting about these situations they would be better equipped to deal with them in the future, in their “life as clerks, or even resident or attending physician.” (No. 72)

*This test led me to feel a little more at ease about the future, if ever I should be confronted with similar situations.* (No.99)

Another theme that emerges from the data is that clerks greatly appreciated having access to multiple opinions from experts about each case. They quickly recognized the value of contrasting them in terms of greater wealth of reflection about such issues. In particular, they felt that the divergent opinions reassured them “on the power of their judgment.” (No.72)

*I experienced one of the situations presented and I asked myself what was the best way for me to act. I also liked the answers given by the experts. I found it fantastic that some experts said “totally inacceptable” while others said “somewhat acceptable”. I really liked it. I also liked that this exercise was made available after a few weeks of clerkship, it allowed me to handle difficult experiences and to appreciate the relevance of the exercise. I think just one [LbC] session is sufficient (it allows for confidence, especially when we see that there is not just one correct answer, without it being too much).* (No.89)

Clerks recognized that, as far as professionalism is concerned, there is not one single answer and grey zones are part of medical practice. These subtleties made them aware that there is no “right answer.”

*It is also very difficult to aim for the exact answer often considering the possible answers (totally, partially, a little, etc.). But the test is still very relevant if we take the time to give a full answer (not only by clicking on one answer option) and comparing this response with that of experts.* (No.21)

Clerks readily recognized the added value of LbC as compared to theoretical courses “for once, I find that it’s fun and educational to do this kind of exercise.”(No.106) This denotes a preference for being exposed to ethical principles through concrete situations rather than through lectures and readings. One student was surprised by how enjoyable learning about professionalism was with this tool, which can otherwise be “lengthy and non-stimulating.” (No.70) One clerk singled out how useful the built-in detailed feedback was:

*This LbC allowed me to reflect on delicate situations and get an immediate feedback, which is not always the case when confronted with this type of situation.* (No.93)

Clerks did not fail to recognize that the important aspect of the tool was the justification given for the answers. They appreciated being exposed to divergent expert opinions, but appreciated having some guidance, by the way of the synthesis expressed by panel members.

*The synthesis (key points to remember) for each item is greatly appreciated, considering the great variability of opinion among experts.* (No.129)

*I think it is good to include the comments of those who have responded in writing, but especially the “wrap up” summary of the general idea of the answers.* (No.62)

In summary, clerks found that the situations presented in the concordance of judgment tool were realistic, and relevant for their clerkship and future practice.

## Discussion

These three examples of LbC show some degree of convergence as to how students adapt and recognize its value for learning. The lack of clinical exposure of 1st year and 2nd year (pre-clinical) students (as mentioned by the students themselves) didn’t prevent them from finding LbC helpful for learning and relevant for their future practice. Many students in hematology mentioned that it was easier to acquire this knowledge from the LbC tool than from the course textbook.

Another notable result is students’ appreciation for the tool’s embedded subtleties that reflect medical practice, where decisions hinge very often on the difference between “totally acceptable” and “somewhat acceptable.” Comments provided by students show that they reacted positively to being forced to position themselves about the uncertainty in the situation. Albeit 1^st^ year students found it hard to accept that there was no one right answer, 3^rd^ year students very much appreciated the nuances separating expert opinions. Furthermore, although 1^st^ year students found the clinical scenarios difficult due to little clinical exposure, they recognized that LbC helped them identify gaps in their knowledge and allowed them to focus their study to better prepare for exams. It would seem therefore that early introduction to uncertainty and ambiguity in medical studies is possible and may even be desirable.

Students also promptly recognized the learning potential of being able to compare their reasoning processes with those of the experts. In the three LbC cases presented here, students mentioned that having access to expert explanations was greatly appreciated and useful for their learning. This is consistent with a meta-analysis conducted by Wang *et al.*^19^ on variables that influence learning where it was found that the quantity of instruction and classroom interactions were amongst the most important. Finally, the results highlight the importance of the role of the instructor– as role model – in providing meaning and structure to learning as suggested by the Cognitive Apprenticeship models.^1^,^2^ A key feature of LbC is that experts, by providing explanations for their answers, are in effect modeling their reasoning skills for the benefit of their students. 3,4

### Limitations

An initial overarching limit is that the findings rest on student perceptions written shortly after having completed the LbC exercise. They are focused on their recent experience with the tool rather than on the long-term learning that it may support.

Moreover, there is a desirability bias. Students know that their instructors and faculty officials will likely read their comments. As a way to limit this bias, we left aside the congratulatory and encouraging comments and focused on the comments that offered a critical opinion of which there were many. On the other hand, students were not prompted about what to write in their comments except a vague invitation to leave a comment. Hence, they freely volunteered information about their experience with LbC. We conclude that themes that emerged in our analysis were sufficiently salient in students’ minds for them to write their comments.

### Conclusion

The increase demand on Health Services worldwide notwithstanding, medical training is becoming more and more complex. It is sufficient to see the competency frameworks being imposed on medical schools to realize that new doctors must learn and be proficient on many more competencies than before, without a substantial increase in teaching resources. Hence, there is a marked trend to find effective and less-resource intensive means to train physicians. There is a case to be made for on-line LbC, which blends cognitive apprenticeship, just-in-time learning and low costs. Web-based learning platforms using LbC facilitate student access to the course material and to highly contextualized expert feedback. Methodologies have been developed to assist instructors in designing and operating LbC tools for their courses.

The results presented here lend credence to the idea that LbC may be an effective way to acquire contextualized knowledge to support transition from theoretical courses in the pre-clinical years to clinical practice. They are consistent with cognitive apprenticeship claims that by purposely revealing their mental processes, instructors provide effective support for problem solving skills development in students. Hence, exposing undergraduate students to uncertainty and ambiguity of clinical practice is possible; students find it challenging but they recognize that this is what their future practice will be like. Further investigation is required about the long term impacts of LbC. We have only just begun to use LbC in pre-clinical medical studies and instructors and faculty administrators need to be provided evidence that LbC is an effective learning tool for a range of competencies. Studies in which 1^st^ year cohorts who use LbC can be followed and compared with cohorts who don’t use LbC on a longitudinal basis would be a fruitful next step.

## Figures and Tables

**Table 1:** Screen content that participants successively discover (LbC Clinical Reasoning, 1^st^ year students, Haematology course)

**Clinical Case:** A 40 year-old female patient presents herself in your office. Blood tests reveal microcytic anemia.		
1st screen		
**If you are thinking of …** Iron absorption anemia	**And then you find…** That a duodenal biopsy shows atrophy of intestinal villi	**The effect of this new information on your initial thought is:** Positive □ Neutral □ (doesn’t change) Negative □
2nd screen		
**Instructors’ response (n=2)** (1st feedback source)		Positive
3rd screen		
**Key-Message:** (2nd source of feedback) The duodenum and proximal jejunum play an important role in the iron absorption. Any disease affecting the duodenum, such as celiac disease, impedes the absorption of iron and increases the risk of developing iron deficiency anemia. Among the various aetiologies of iron deficiency, one must consider: 1- blood loss (especially gastrointestinal or gynaecological), 2- iron absorption anomaly (as in this case) and 3- inadequate iron intake.		

**Table 2:** Series of screens that the participant discovers (Concordance-of-Professional-Judgment)

**Situation:** Your resident has prescribed penicillin for a hospitalized patient for an infection. Consulting the patient digital file, you see that it clearly states that the patient is allergic to penicillin. The patient has received two doses before you noticed the error and is asymptomatic.	
1st screen	
**Once you pointed out the error, the resident…** ... changes the prescription, but doesn’t say anything to the patient.	**This attitude is...** Totally inacceptable □ Hardly acceptable □ Somewhat acceptable □ Totally acceptable □
2nd screen	
**Panel responses** (n=8) (1st source of feedback)	Totally inacceptable 7/8 Hardly acceptable 1/8
3rd Screen (2nd source of feedback)	
**Explanations given by panel members** Member 1: It is necessary to inform the patient so that he is aware that he is not allergic to penicillin. By revealing the situation, he will be able to inform other doctors that he is not allergic. Most patients react favorably when an error is revealed to them. Member 2: The patient has a right to know and the doctor has the obligation to inform the patient of the incident, as it is stated in the Code of Ethics of the Quebec College of Physicians. Member 3: It’s transparency issue. The patient has a right to know and this transparency is usually very beneficial for the patient-doctor relationship. Member 4: This is a case of FALSIFYING the patient file. This is: probably illegal shows a flagrant lack of ethical concern This incident must be used to show the resident’s lack of ethical standards, by using it as a learning opportunity and it should be noted in the resident’s academic record. Member 5…	
4th screen	
**Key Messages (3rd source of feedback)** This case is about the reporting of errors. Article 56 of the Deontological Code states: *The Physician must inform, as soon as possible, his patient or his legal representative, of any incident, accident or possible complication likely to bring about or to have brought about significant consequences on the state of his health or his physical integrity.* In the above situation this means that the resident must tell the patient what has happened. This is a case where telling the truth is a formal obligation. While discussing with the patient, he must avoid using the word “error” but speak instead of undesirable event because it is up to legal authorities to determine whether there was an error or lack of due diligence. It is up to the physician responsible for the undesirable event to inform the patient. The role of colleagues is not to denounce but to provide support for reporting the error. The vast majority of patients will react positively and collaboratively to open and frank discussion during the reporting of the error and will participate in the identifying and implementing of the corrective actions. Patients will often react badly and with anger if they learn about the situation from indirect sources. The golden rule in error reporting is to ensure quality communication and show utmost respect for the patient.	

**Table 3:** Study population

	Class	Setting	Number of cases / questions	Sample sizes

Concordance of reasoning	1^nd^ Year Haematology-oncology course	In class on a common computer (PBL session)	1 case with 4 questions in each of the 6 PBL sessions	300*/171**/58***
Concordance of perception	2^nd^ Year Pulmonary physiopathology course	Voluntary exercise completed on student’s personal computers (PBL course)	10 sets of images	300* / 241** / 199***
Concordance of professional judgment	3^st^ year Clerkship	Voluntary exercise completed on student’s personal computers	20 cases	300* /241**/147***

* Cohort size /

** Number of students who took the LbC activity /

*** Number of quantitative responses analyzed
